# Improving the Bioactivities of Apricot Kernels Through Fermentation: Investigating the Relationship Between Bioactivities, Polyphenols, and Amino Acids Through the Random Forest Regression XAI Approach

**DOI:** 10.3390/foods14050845

**Published:** 2025-02-28

**Authors:** Zhiyu Zhao, Kevin Kantono, Rothman Kam, Thao T. Le, Eileen Kitundu, Tony Chen, Nazimah Hamid

**Affiliations:** AUT Centre for Future Foods, School of Science, Faculty of Health and Environment Sciences, Auckland University of Technology, Auckland 1010, New Zealand; nmk0840@autuni.ac.nz (Z.Z.); kkantono@aut.ac.nz (K.K.); rothman.kam@aut.ac.nz (R.K.); thao.le@aut.ac.nz (T.T.L.); eileen.kitundu@aut.ac.nz (E.K.); tony.chen@aut.ac.nz (T.C.)

**Keywords:** apricot kernels, fermentation, lactic acid, amino acids, antioxidant activity, polyphenols, random forest regression

## Abstract

Apricot kernels are known for being a rich source of oil, protein, and bioactive compounds. This study focused on enhancing the bioactivities of apricot kernels through fermentation. Additionally, this study explored the correlations between polyphenols, amino acids, antioxidant activities, and total phenolic content (TPC). The findings indicated that apricot kernels fermented with *Lactiplantibacillus plantarum* exhibited increased antioxidant activity, as assessed by the FRAP and CUPRAC methods, and an increased TPC compared to naturally fermented samples. The CUPRAC activity increased significantly from 1.03 to 1.82 mg of ascorbic acid per gram of sample on day 7, and the FRAP activity increased from 4.9 to 12.2 mg of ascorbic acid per gram of sample on day 3 of fermentation. Moreover, the TPC significantly increased from 1.67 to 7.58 mg of gallic acid per gram of sample on day 9 of fermentation. The results further demonstrated that, during the fermentation process, the concentration of hydroxybenzoic acid increased from 0.52 µg/g on day 0 to 5.3 µg/g on day 9. The DL-3-phenyllactic acid content demonstrated a significant increase from 0.42 µg/g on day 0 to 99.62 µg/g on day 5, while the benzoic acid content exhibited a notable increase from 45.33 µg/g to 138.13 µg/g over the fermentation period, with peak levels being observed on day 5. Similarly, most amino acids demonstrated a rise in concentration as the fermentation progressed, peaking on the ninth day. This study further employed random forest regression as a form of explainable artificial intelligence (XAI) to explore the relationships between phenolic compounds, amino acids, and antioxidant activities. Amino acids like L-cystine and L-anserine were found to positively impact FRAP values, while L-histidine and 1-methyl-L-histidine contributed to the CUPRAC antioxidant activity. Notably, hydroxybenzoic acid emerged as a key contributor to both the FRAP value and TPC, highlighting its significance in improving the overall antioxidant capacity of apricot kernels. These findings indicate that, under optimised fermentation conditions, apricot kernels hold promise as functional food ingredients due to the beneficial antioxidant properties observed in this study.

## 1. Introduction

Apricot (*Prunus armeniaca* L.), a species of the Rosaceae family, is recognised for its health-enhancing attributes, which are due to its rich composition of vitamins, flavonoids, and potassium. Apricot kernels also offer potential health benefits, being rich in healthy fats, protein, and fibre. Approximately 50% of their dry weight is comprised of oil, with monounsaturated fats such as oleic acid making up around 60–73% of the total oil content, and polyunsaturated fats like linoleic acid accounting for 24–37% of the same [1]. Additionally, apricot kernels are a valuable source of high-quality protein, with their protein content ranging from 14.1 to 45.3% [2].

The apricot kernel possesses antioxidant and antimicrobial activities. It is a rich source of vitamins, particularly vitamin E, with levels ranging from 0.003 to 0.040 g/100 g, [3] which aids in protecting cells from oxidative damage. Additionally, the presence of various polyphenolic compounds, such as anthocyanins, phenolics, flavonoids, and carotenoids [4], contributes to their antioxidant and anti-inflammatory properties. Research by Lima et al. [5] revealed that apricot kernel extracts significantly inhibited the proliferation of various microbial species, particularly *Staphylococcus aureus* and *Salmonella*. Akhone, Bains [4] highlighted that phenolic compounds are the primary contributors to the bioactive properties of apricot kernels.

Numerous studies have shown that microbial fermentation, especially by lactic acid bacteria (LAB), significantly enhances the antioxidant activity of foods in various dietary matrices [6]. For instance, De Montijo-Prieto et al. [7] discovered that the FRAP antioxidant activity of avocado puree fermented with *Lactiplantibacillus plantarum* progressively increased, reaching a maximum at 48 h. Similarly, Mohapatra et al. [8] observed a significant enhancement in the DPPH scavenging activity and CUPRAC activity of sorghum following 48 h of LAB fermentation. Furthermore, Đorđević et al. [9] found that the fermentation of buckwheat, wheat germ, barley, and rye by *Lactobacillus rhamnosus* produced superior antioxidant activity compared to fermentation by *Saccharomyces cerevisiae*. Kuligowski et al. [10] noted that the polyphenol levels in soybeans reached their zenith after four days of fermentation with the *Rhizopus oligosporus* strain, suggesting that fermentation liberates bound phenolic components, thus enhancing antioxidant activity [11]. Additional research also indicates that LAB fermentation enhances the antioxidant capacity of soy germ [12], soybean [13], and lentils [14]. The metabolism of phenolic compounds during LAB fermentation is physiologically important, and the resulting metabolic by-products may offer potential health benefits for humans [15].

Machine learning methods have been employed to investigate the relationship between dietary metabolites and antioxidant activity. A combination of UPLC-QTOF/MS-based metabolomics and three machine learning algorithms was utilised to distinguish various cherry species based on their TPC, total flavonoid content (TFC), and antioxidant activity [16]. The research indicated that Prunus tomentosa exhibited elevated TPC and TFC, along with enhanced antioxidant activity, with flavonoids being recognised as possible factors influencing these variations. A separate study analysed the phenolic profiles and antioxidant activities of free and bound fractions from extracts of eight blackberry varieties using boosted regression trees, identifying cyanidin-3-O-glucoside and chlorogenic acid as the primary compounds contributing to the antioxidant activity of the free fractions [17].

Apricot kernels possess antioxidant and antimicrobial activities and are a rich source of vitamins (particularly vitamin E), healthy fats, protein, and fibre. They also contain polyphenolic compounds that contribute to their antioxidant and anti-inflammatory properties. Numerous studies have demonstrated that microbial fermentation, especially by lactic acid bacteria (LAB), can significantly enhance the antioxidant activity in various cereals and legumes. The bioactivity enhancement of apricot kernels through controlled fermentation remains unexplored. This study innovatively combines fermentation with machine learning to understand the relationships between metabolites and bioactivities, providing a novel framework for developing apricot kernels as functional food ingredients. Hence, the objective of this research is to enhance the bioactivities of apricot kernels through fermentation and to investigate the relationships between their polyphenols, amino acids, antioxidant activities, and total phenolic content (TPC). This study uses random forest regression and SHAPs analysis to identify key compounds that are responsible for antioxidant activities.

## 2. Materials and Methods

### 2.1. Fermentation Process

Apricot (*Prunus armeniaca*, Sundrop variety) kernels were sourced from Waitaki Orchards Ltd., Otago, New Zealand. The fermentation process, based on Zhao et al. [18], included some modifications. A natural fermentation control group (no LAB inoculum) was included in this study. Each batch used 100 g of apricot kernels for both *L. plantarum* fermentation and natural fermentation. A 1% *L. plantarum* solution was prepared by combining 1 mL of a *L. plantarum* stock culture with an approximate bacterial suspension of 9.0 × 10^8^ CFU/mL (equivalent to a 3.0 McFarland standard) in 100 mL of MRS broth. The inoculated solution was then incubated at 37 °C for 24 h and subsequently used to inoculate the apricot kernels, which were then incubated in a carbon dioxide incubator under anaerobic conditions at 37 °C for 9 days. Microbial counts were performed as described previously, with additional plating on Acetobacter and Malt Extract agar for natural fermentation samples. Both *L. plantarum*-fermented and naturally fermented apricot kernels were freeze-dried (Christ alpha 2–4, Marin Christ, Osterode am Harz, Germany) for 48 h, and then ground into powder. The freeze-dried samples were stored at −20 °C until analysis.

### 2.2. Sample Extraction

#### 2.2.1. Preparation of Sample Extract for Antioxidant Analysis

The methodology for preparing sample extracts for antioxidant analysis was modified from that in Zhao et al. [18] to be specific to apricot kernels. In summary, 0.1 g was extracted from both fermented and raw powdered apricot kernel samples using 4 mL of pure methanol, following which it underwent homogenisation and centrifugation. The methanol extraction was conducted again, and the resultant supernatant was diluted to a final amount of 10 mL using methanol. The resultant extract (S1) was preserved at −20 °C for future TPC, CUPRAC, and FRAP tests. Methanol was chosen for TPC, CUPRAC, and FRAP assays due to its high efficiency in extracting polar (such as phenolic acids) and semi-polar compounds, ensuring comprehensive recovery of antioxidants [19].

#### 2.2.2. Preparation of Sample Extract for Polyphenol and Lactic Acid Analysis

Each ground apricot kernel sample (0.25 g) was weighed in an Eppendorf tube and sonicated in 1 mL of 80% ethanol–water mixture for 30 min according to Khan et al. [20]. This sample extract (S2) was produced for the study of polyphenol and lactic acid levels because ethanol is an excellent solvent for polyphenols, and the addition of water likely enhances the extraction of organic acid (such as lactic acid), which is highly soluble in water [21,22].

#### 2.2.3. Preparation of Sample Extract for Amino Acid Analysis

Ground raw and fermented apricot kernel (0.02 g) samples were weighed and placed in an Eppendorf tube. Due to the high fat content of apricot kernels, ranging from 40.2% to 50.9%, a defatting process was employed to prevent the interference of fat in the extraction and subsequent analysis of amino acids, which would otherwise result in inaccurate assay results. N-hexane (200 μL) was added to the ground apricot kernels, vortexed for 30 s, and allowed to stand for 5 min. The mixture was then centrifuged at 10,000× *g* for 10 min at 10 °C, and the supernatant was discarded. This defatting step was repeated twice with fresh N-hexane to ensure complete removal of any remaining fat from the sample.

The defatted residue was mixed with 200 μL of 60% acetonitrile and ultrasonicated in an ice bath for 30 min. After centrifugation at 10 °C, the residue was removed, leaving behind 200 μL of supernatant. Then, 60 μL of the sample was incubated on ice for 30 min and subsequently centrifuged at 10,000× *g* for 15 min at 4 °C to remove any existing protein. The supernatant (S3) was immediately stored at −20 °C, analysed within 24 h to prevent degradation, and prepared for AccQ tag derivatisation for LC-MS analysis.

### 2.3. Antioxidant Activity

All antioxidant analyses, including TPC, CUPRAC, and FRAP analyses, were performed according to the methodologies outlined by Zhao et al. [18]. TPC was quantified at 765 nm utilising the Folin–Ciocalteu test, with the results reported as mg of gallic acid equivalents per gram of sample. The CUPRAC and FRAP experiments were conducted at 450 nm and 593 nm, respectively, utilising ascorbic acid standards, with the findings reported as mg of ascorbic acid equivalents per gram. All samples were analysed in triplicate.

### 2.4. Polyphenols in Fermented Apricot Kernels

The polyphenolic composition of apricot kernel samples was analysed following Liu et al. [23] with minor adjustments. LC-MS analyses were conducted using an Agilent 1260 Infinity Quaternary LC System connected to a 6420 mass spectrometer an with electrospray ionisation source (Santa Clara, CA 95051 USA). The separation of polyphenols was performed on an Agilent Poroshell 120 EC-C18 column (2.1 × 100 mm, 2.7 µm) with a flow rate of 0.25 mL/min and column temperature of 25 °C.

The mobile phase consisted of (A) HPLC grade water + 0.1% formic acid and (B) acetonitrile + 0.1% formic acid. A linear gradient program was used: 97% A/3% B at 0 min and held for 0.5 min; 85% A/15% B at 0.5–1 min; 75% A/25% B at 1–6 min; 65% A/35% B at 6–8 min; 50% A/50% B at 8–9 min and held for 2 min; 20% A/80% B at 11–12 min and held for 1 min. The total run time was 23 min. The conditions for the MS analyses were as follows: drying gas (N_2_), temperature at 300 °C, drying gas flow at 10 L/min, nebuliser pressure at 40 psi, and capillary voltage of 4 kV. The negative ionisation mode was performed with MRM for quantitative analysis. The MRM transitions are as shown in the Supplementary Materials (Table S1). Standards utilised for the quantification of individual phenolics in fermented apricot kernels include hydroxybenzoic acid, lumichrome, chlorogenic acid, DL-3-phenyllactic acid, catechin, benzoic acid, p-coumaric acid, quinic acid, caffeic acid, epicatechin, and quercetin, all of which were used for comparison and identification purposes.

### 2.5. Free Amino Acid Analysis

The AccQ-Tag method was performed as described by Salazar et al. [24]. Briefly, 40 µL of either the sample acquired in Section 2.2.3 or the calibration standard was mixed with 40 µL of methanol containing d4-Alanine (10 µg/mL), vortexed, and then centrifuged at 11,290× *g* for 5 min at 4 °C. Next, 10 µL of the supernatant was added to 70 µL of 0.2 M borate buffer, followed immediately by 10 µL of 0.01 M AccQ-Tag (6-aminoquinolyl-N-hydroxysuccinimidyl carbamate) solution, and the mixture was vortexed. The solution was incubated at 55 °C for 15 min. Subsequently, 400 µL of 10% formic acid was added before the mixture was transferred to an autosampler vial for LC-MS analysis. Calibration standards (200–1.5625 µM) were prepared by serial dilution of a 500 µM stock of A9906 physiological amino acid standards purchased from Sigma-Aldrich (Auckland, New Zealand). Standards utilised for the quantification of individual amino acids in fermented apricot kernels include L-histidine, 1-methyl-L-histidine and 3-methyl-L-histidine, hydroxy-L-proline, L-arginine, asparagine, taurine, glutamine, L-serine, ethanolamine, glycine, L-aspartic acid, L-citrulline, L-glutamic acid, b-alanine, L-threonine, L-alanine, γ-amino-n-butyric acid, L-proline, L-ornithine, L-α-amino-n-butyric acid, L-lysine, L-cystine, L-anserine, L-tyrosine, L-methionine, L-valine, L-isoleucine, L-leucine, L-phenylalanine, and L-tryptophan.

LC-MS analyses were conducted using an Agilent 1260 Infinity Quaternary LC System connected to a 6420 mass spectrometer with an electrospray ionisation source (Santa Clara, CA 95051 USA) [25]. The column used was a Kinetex Evo C18 (2.1 × 150 mm, 1.7 µm) (Phenomenex, Torrance, CA, USA).

The MS ionisation source conditions were as follows: capillary voltage of 4 kV, drying gas temperature of 300 °C, drying gas flow of 10 L/min, and nebulizer pressure of 30 psi. The positive ionisation modes were performed with multiple reaction monitoring (MRM) for quantitative analysis. The MRM transitions are shown in the Supplementary Materials (Table S2). The LC conditions were as follows: Mobile phase A was 0.1% formic acid in UPW. Mobile phase B was 0.1% formic acid in acetonitrile. The flow rate was 0.25 mL/min. The gradient program was as follows: The initial percentage of mobile phase B (B%) was 5%, which was held for 3 min. B% was then raised at a rate of 1% per minute to 15%. B% was then raised to 80% in 3.5 min, and held for 0.5 min. B% was then lowered at a rate of 50% per minute to 5%, and held for 10.5 min, at which point the run was complete.

### 2.6. Lactic Acid and Amygdalin Content

#### 2.6.1. Lactic Acid

The lactic acid content of the fermented apricot kernel sample was analysed according to Chessum et al. [25]. For qualitative and quantitative analysis of lactic acid, the calibration curves for lactic acid showed excellent linearity over the range of 0.078125–40 mg/mL (R^2^ = 0.999, n = 9), and LC-MS analyses were conducted using an Agilent 1260 Infinity Quaternary LC System connected to a 6420 mass spectrometer with an electrospray ionisation source (Santa Clara, CA 95051 USA). MRM scan was configured with a precursor/product ion m/z of 89/43. Lactic acid was isolated using a Kinete EVO C18 column (2.1 × 150 mm, 1.7 µm) at 25 °C with a flow rate of 0.20 mL/min, under conditions analogous to those employed in polyphenol analysis. A linear gradient program was used with the following specifications: starting point was 100% A/0% B, which was held for 0.2 min, after which the ratio was shifted to 80% A/20% B from 0.2 to 1 min to 50% A/50% B from 1 to 7 min, and finally back to 100% A/0% B from 7 to 10 min. The total runtime was 25 min.

#### 2.6.2. Amygdalin

The amygdalin extraction method was modified from Bolarinwa et al. [26]. The fermented apricot kernel samples were freeze-dried and pulverised into a powder. A combination of 2 g of the powder and 50 mL of 100% ethanol was subjected to boiling for 100 min. Following the attainment of room temperature, the extract was subjected to filtration. Subsequently, a rotary evaporator was utilised to completely evaporate the ethanol at 35 °C and 7 mbar. Following that, 10 mL of diethyl ether was added to the dried sample, which was then vortexed for 1 min at ambient temperature to facilitate the precipitation of amygdalin. The amygdalin precipitate was dissolved in 5 mL of Milli-Q water, and 1.5 mL of the solution was transferred to an Eppendorf tube. Centrifugation was conducted for 10 min at 22 °C and 11,290× *g*, followed by filtration through 0.45 µm PTFE filters into an autosampler vial, in preparation for subsequent LC-MS analysis.

The LC-MS analysis was performed according to the set-up described in Permal et al. [27]. Quantification was performed using commercial amygdalin (99.12% purity) standard to generate a calibration curve in the range of 0.0195–10 mg/L (R^2^ = 0.9993). LC-MS analyses were conducted using an Agilent 1260 Infinity Quaternary LC System connected to a 6420 mass spectrometer with an electrospray ionisation source (Santa Clara, CA 95051 USA).

Phenomenex Kinetex Evo C18 (2.1 × 150 mm, 1.7 µm) column was used for analysis of amygdalin. The mobile phases were composed of water containing 0.1% (*v/v*) formic acid (A) and acetonitrile containing 0.1% (*v/v*) formic acid (B). The initial gradient conditions were 95:5 (A:B). From 1 to 2 min, B was increased to 12%. From 2 to 6 min, B was increased to 20%. From 6 to 8 min, B was increased to 90%, which was then held for 3 min. From 12 to 13 min, B was decreased to 5%. The injection volume was 3 µL and the total run time for each sample was 23 min.

The MS ionisation source conditions were set to a capillary voltage of 4 kV and corona current of 4 µA. Drying gas (N2) temperature of 300 °C at a flow rate of 10 L/min and nebuliser pressure of 30 psi was used. A positive ion mode was used with MRM for quantitative analysis. Precursor-to-product ion transition used for amygdalin was [M + H] + m/z 456 → 323 (quantifier ion), 221 (qualifier ion) with a fragmentor voltage of 220 V, and collision energy of 3 eV and 8 eV, respectively. The relative abundance was 17%, within the 20% acceptance criteria.

The matrix effect can be calculated using the method described in Panuwet et al. [28], and it was calculated as follows:

$$ME(\%)=\frac{{Area}_{spiked\;post-extracted\;matrix}}{{Area}_{spiked\;solvent\;solution}}$$

The matrix effect was calculated at 98.4%, showing 1.6% ionization suppression. According to FDA guidelines, an ME within 80–120% is acceptable without matrix compensation. Furthermore, our method validation demonstrated high accuracy (97.5–102.3%), confirming that the minimal ME did not affect performance. The chromatographic repeatability (n = 5) was determined and the residual standard deviation (RSD), limits of detection (LODs), and limit of quantification (LOQ) were calculated as outlined by Permal et al. [29].

$$\mathrm{LOD}=\frac{3.3\;\times \;S_{residuals}}{\mathrm{Slope}}$$

$$LOQ=\frac{10\times S_{residuals}}{Slope}$$

1. S_residuals_: standard deviation of the residuals error of the calibration curve;

2. Slope: slope of the calibration curve;

3. RSD, LOD, and LOQ were 10.5%, 0.05 µg/mL, and 0.15 µg/mL, respectively.

### 2.7. Statistical Analysis

#### 2.7.1. Microbial, Antioxidant and Total Phenolic Content

All statistical analyses were performed using XLSTAT 2024 (Lumivero, Denver, CO, USA). For microbial growth and antioxidant activity (TPC, CUPRAC, FRAP), a two-way ANOVA with random effects of samples (L0, L1, etc.) was applied to evaluate the fixed factors of fermentation method (*Lactiplantibacillus plantarum* vs. natural fermentation) and time (from day 0 to day 9). Post-hoc comparisons were conducted using Fisher’s LSD test at a significance level of *p* < 0.05.

#### 2.7.2. Amino Acids Analysis

The heatmaps were produced using the MetaboAnalyst 5.0 software (www.metaboanalyst.ca, accessed on 8 September 2024, Canada), which provides a visual representation of the changes in concentration of specific amino acids and phenolic compounds for different fermentation times and bacterial conditions. The clustering dendrograms provided additional insight by revealing the relationships between the variables and identifying clusters of compounds that exhibited similar behaviour under the experimental conditions. To ensure comparability across variables, the data were normalised and auto-scaled. The Euclidean distance was selected as the similarity measure to quantify the geometric distance between samples, effectively capturing the overall differences in concentration levels among the variables. For grouping of samples and compounds, the Ward’s linkage method was used as the clustering algorithm. This method minimised the variance within each cluster, resulting in more cohesive groupings.

### 2.8. Multiple Factor Analysis (MFA)

To analyse the correlations between observations and variables in this study, a proficient method known as MFA, which enables simultaneous analysis of multiple variable datasets, was utilised [30]. Using XLSTAT 2024 (Lumivero, Denver, CO, USA), the investigation aimed to explore how levels of polyphenols, amino acids, and lactic acid were correlated with the various antioxidant activities observed across all fermented apricot kernel samples during the fermentation process. This study employed MFA to investigate the correlation among polyphenols, amino acids, and antioxidant activity.

### 2.9. Machine Learning

In this study, an unsupervised machine learning technique was used to analyse the data. Specifically, random forest (RF) regression combined with Extreme Gradient Boost (XGBoost) [31] was employed. To optimise the performance of the models, hyperparameters such as gamma, learning rate, maximum tree depth, minimum child weight, and estimators were tuned using the GridSearchCV technique [32]. Furthermore, the SHapley Additive exPlanations (SHAPs) values were extracted to examine the contribution of phenolic compounds and amino acids to the antioxidant activity. All machine learning analyses were conducted using Python 3.10.6 (Python Software Foundation, Beaverton, OR, USA). Machine learning (ML) XGBoost was used to describe the relationship between the amino acids, polyphenols, FRAP, and TPC.

## 3. Results and Discussion

### 3.1. Microbial Growth

The fermentation led to significant changes in the LAB counts, with peaks observed on day 5 in the *L. plantarum*-fermented samples and on day 7 in the naturally fermented samples. Detailed data are provided in Supplementary Section S1.

### 3.2. Antioxidant Activities

As shown in Table 1, significant variations were observed in the TPC, CUPRAC, and FRAP antioxidant activities within the naturally fermented and *L. plantarum*-fermented apricot kernel samples during the fermentation process. Specifically, the naturally fermented samples displayed notably lower levels of TPC, CUPRAC, and FRAP activities.

#### Changes in Antioxidant Activity

The antioxidant activities of the fermented apricot kernels, assessed via CUPRAC and FRAP assays, exhibited different trends over time, being influenced by the fermentation methods as shown in Table 1. In the *L. plantarum*-fermented samples, the CUPRAC values increased progressively from 1.03 to 1.82 mg ascorbic acid/g sample from day 0 to day 7. While the CUPRAC results demonstrated a consistent increase in antioxidant activity over the fermentation period, the FRAP results presented a different pattern. The FRAP activity of the *L. plantarum*-fermented samples peaked earlier at 12.20 mg ascorbic acid/g sample on day 3, followed by a gradual decline (Table 1). CUPRAC primarily detects phenolic-driven electron transfer, such as that mediated by hydroxybenzoic acid, while FRAP reflects the iron reduction capacity enhanced by sulphur-containing amino acids such as L-cystine.

The high CUPRAC activity in the apricot kernels fermented by *L. plantarum*, reaching 1.82 mg ascorbic acid/g sample compared to 0.81 mg ascorbic acid/g sample in natural fermentation, corroborates the findings by Mohapatra et al. [8], who reported a six-fold CUPRAC increase in LAB-fermented sorghum. This trend is further supported by the research of Adetuyi et al. [33], which showed a significant increase in the phenolic content, vitamin C content, total flavonoids, and non-flavonoid compounds, as well as increased antioxidant activity, in fermented okra seeds relative to unfermented samples. However, the FRAP peak observed on day 3 of fermentation contrasts with the study by De Montijo-Prieto et al. [7], where avocado puree fermented by *L. plantarum* showed sustained FRAP increases until 48 h. This discrepancy may stem from substrate-specific metabolite dynamics. The high lipid content of apricot kernels likely accelerates the oxidative degradation of labile antioxidants such as epicatechin, thereby shortening the time of FRAP efficacy.

The natural fermentation minimally enhanced the CUPRAC activities, with values ranging from 0.67 to 0.81 mg ascorbic acid/g sample, but moderately increased the FRAP levels, for which the values increased from 4.51 to 7.71 mg ascorbic acid per gram sample. This suggests that native microbes in apricot kernels preferentially generate iron-reducing metabolites such as organic acids over phenolic antioxidants. This result corresponds with the findings by Chinma et al. [34], who found that naturally fermented rice bran flour demonstrated a significantly reduced antioxidant capacity in comparison to yeast-fermented variants. Moreover, Othman et al. [35] demonstrated that the Trolox equivalent antioxidant capacity (TEAC) values of spontaneously fermented green, varicoloured, and black olives showed a higher reduction compared to samples that underwent inoculated fermentation (*L. plantarum*). These findings highlight the unique ability of *L. plantarum* to enhance both pathways, positioning it as a superior starter for functional ingredient development.

### 3.3. Total Phenolic Content (TPC)

The two-way ANOVA results presented in Table 1 demonstrated changes in the TPC levels across different fermentation times. In the *L. plantarum*-fermented samples, the TPC increased significantly from day 0 to days 3–5, decreased on day 6, and then peaked on day 9 of fermentation. This reflects the complexity of phenolic compound biosynthesis during the fermentation process. Adetuyi et al. [33] observed that fermented okra seeds exhibited a higher TPC compared to raw seeds, with their levels increasing from 185 to 385 mg GAE/100 g after 120 h of fermentation. Similarly, Cheng et al. [36] found that black soybeans fermented by *Rhizopus oligosporus* and *R. oryzae* at 30 °C for 6 days yielded a TPC value of 2876.2 μg/g, which was 1.16 times higher than that in the unfermented sample. Ayyash et al. [37] reported a notable elevation in the TPC levels in lupin, quinoa, and wheat fermented by *L. plantarum*. This increase in TPC levels can be attributed to the utilisation of polyphenols as substrates during fermentation, where they are broken down by polyphenol-associated enzymes into smaller phenolic compounds with enhanced bioactivity and bioavailability [38].

Although the TPC in the naturally fermented samples increased from day 0 to day 9, the increase in the TPC level was much lower than that in the *L. plantarum*-fermented samples. Similarly, in a study by Zhao et al. [18], it was shown that *L. plantarum*-fermented avocado seeds displayed the highest TPC levels in comparison to kefir-fermented and naturally fermented samples.

### 3.4. Polyphenol Analysis

**Figure 1:**
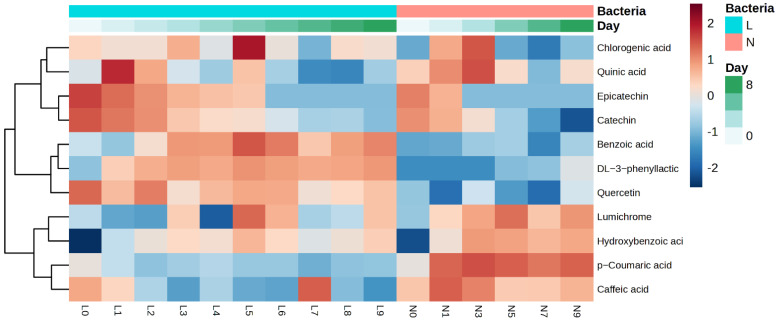
Heatmap and cluster analysis of phenolic compound concentrations (µg/g sample) in naturally fermented and *L. plantarum*−fermented apricot kernels over nine days. The columns in the heatmap represent the phenolic compounds, while the rows represent the fermentation time and methods. (L: L. plantarum fermentation and N: natural fermentation) and days of fermentation (0−9).

Figure 1 shows that the levels of hydroxybenzoic acid, chlorogenic acid, DL-3-phenyllactic acid, benzoic acid, and lumichrome all showed a significant increase, reaching peak levels on the fifth day, followed by a subsequent decline.

Table S3 presents the impact of the natural fermentation and *L. plantarum* fermentation of apricot kernels on their phenolic compound profiles over a nine-day period. The levels of hydroxybenzoic acid, catechin, epicatechin, DL-3-phenyllactic acid, and benzoic acid were significantly affected by the fermentation method and time. As shown in Figure 1, the concentration of epicatechin in the naturally fermented and *L. plantarum*-fermented samples decreased with an increase in the fermentation time, reaching undetectable levels on days 6 and 3, respectively. A comparable pattern was noted for catechin in the *L. plantarum*-fermented samples, although the concentration remained higher than that in the naturally fermented sample. As demonstrated in Table S4, there was a decline in the catechin and epicatechin levels from 4.248 to 0.328 µg/g and from 0.129 to 0 µg/g, respectively. This decline is consistent with the enzymatic activity of *L. plantarum*, which has been shown to preferentially hydrolyse flavanols via β-glucosidase and esterase [39]. This catabolic process generates bioactive metabolites such as DL-3-phenyllactic acid and hydroxybenzoic acid, both of which exhibit enhanced electron-donating capacities [38]. While the degradation of catechin reduces its direct antioxidant contribution, the accumulation of smaller phenolic acids compensates for this loss, as evidenced by the positive correlation between hydroxybenzoic acid and the CUPRAC activity (r = 0.79, *p* < 0.01) [40]. The findings of Starzyńska-Janiszewska et al. [41] also showed a decrease in catechin content during black quinoa seed fermentation. Similarly, in our study, the concentrations of catechin and epicatechin decreased with the duration of fermentation, reaching undetectable levels after 3–6 days of fermentation, depending on the method. The results suggest that *L. plantarum* fermentation can significantly impact the metabolism of these flavanols in apricot kernels. Further studies are needed to determine the enzymes implicated in this metabolism and to identify the resultant metabolites.

Additionally, the concentrations of DL-3-phenyllactic acid and benzoic acid increased significantly with the duration of fermentation (Table S4). The level of DL-3-phenyllactic acid demonstrated a significant increase from 0.42 µg/g on day 0 to 99.62 µg/g on day 5, while that of benzoic acid exhibited an increase from 45.33 µg/g to 138.13 µg/g over the fermentation period, with peak levels being observed on day 5. These compounds were predominantly found in the samples fermented by *L. plantarum*, exhibiting significantly higher levels than those in the naturally fermented sample. In a study by Park et al. [42], it was noted that the peak regions of benzoic acid increased by 64% following *L. plantarum* fermentation. This increase in benzoic acid content can be attributed to the metabolic activities of *L. plantarum* during fermentation. *L. plantarum* is known to possess enzymes that can break down complex organic compounds that are present in the food matrix, leading to the formation of simpler compounds such as benzoic acid. DL-3-phenyllactic acid (3-PLA), an organic acid commonly found in honey and lactic acid bacteria-fermented food, can be produced by various microorganisms, particularly lactic acid bacteria. It has also been revealed that *L. plantarum* possesses enzymes that can convert phenylpyruvic acid (PPA) into 3-PLA [43,44].

Throughout the process of fermentation, a rise in the hydroxybenzoic acid levels was noted, with the spontaneously fermented samples exhibiting slightly elevated concentration compared to those fermented by *L. plantarum*. In the natural fermentation, microorganisms including bacteria and fungi were involved, which enhances the breakdown of macronutrients like carbohydrates and proteins. This breakdown process can result in the generation of various compounds, including hydroxybenzoic acid, as highlighted by Bento-Silva et al. [45]. The higher protein and carbohydrate contents in apricot kernels, ranging from approximately 14.6% to 27.1% and 17.5% to 35.6%, respectively [4], provide a favourable environment for the production of compounds like hydroxybenzoic acid during natural fermentation processes. In contrast, fermentation with *L. plantarum* alone may involve a more limited enzymatic profile, which could result in lower concentrations of hydroxybenzoic acid compared to naturally fermented samples. Tang et al. [46] investigated the impact of lactic acid bacteria fermentation on the level of hydroxybenzoic acid. Their study revealed a decrease in the concentration of quercetin-3-O-rutinose-7-O-α-L-rhamno-side (QRR) post-fermentation, while the hydroxybenzoic acid levels increased compared to the initial stage of fermentation. This observation could be ascribed to the metabolic pathway of QRR, which appears to entail direct cleavage of the C-ring at the C2–O1 and C3–C4 bonds.

The contents of p-coumaric acid and quercetin were impacted solely by the fermentation methods that were employed. The *L. plantarum*-fermented samples exhibited higher quercetin production, while the naturally fermented samples showed an increase in p-coumaric acid levels. Research by Carciochi et al. [47] revealed that the fermentation of quinoa had a significant influence on its p-coumaric acid content. After natural fermentation, the p-coumaric acid content increased by nearly 1.4 times compared to the raw sample. Conversely, the quercetin content decreased to undetectable levels under the same fermentation conditions used to determine the p-coumaric acid content. In contrast, Starzyńska-Janiszewska et al. [41] reported an increase in the quercetin content in quinoa seeds fermented with *Rhizopus oligosporus* for 4 days, from 0.022 to 0.061 mg/100 g dry weight basis.

### 3.5. Free Amino Acids Content Analysis

**Figure 2:**
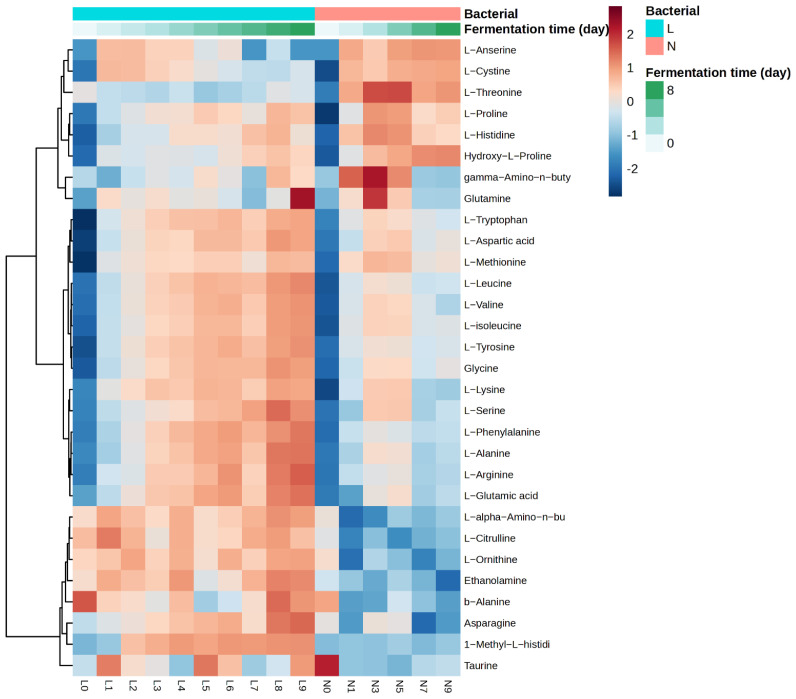
Heatmap and clustering analysis of amino acid concentrations (mg/g sample). The columns in the heatmap represent the different types of amino acid, while the rows represent the fermentation methods (L: *L. plantarum* fermentation and N: natural fermentation) and days of fermentation (0−9).

A comparison of the amino acid contents between the *L. plantarum*-fermented and naturally fermented samples was conducted using heat map analysis (Figure 2), revealing a significant increase in the levels of all amino acids, with the exceptions of taurine and glutamine (Table S5). Table S5 illustrates that the levels of most amino acids increased significantly with the fermentation time in both the *L. plantarum*-fermented and naturally fermented samples, as indicated by the ANOVA results (*p* < 0.001). Nevertheless, the amino acid content remained consistently higher in the *L. plantarum*-fermented samples compared to the naturally fermented samples across the fermentation time. The process of fermentation not only increases the protein value of soybeans from 47.08% to 52.08%, but also improves the availability and absorption of amino acids and minerals in sesame by reducing the levels of anti-nutritional factors such as phytic acid and oxalic acid [48]. This is an essential process which substantially decreases the content of anti-nutrients, such as phytic acid, lectins, tannins, and polyphenols, of legumes [49].

*L. plantarum* fermentation effectively increased the levels of key amino acids compared to those in the naturally fermented samples. Table S3 showed that the levels of most of the essential amino acids, including L-leucine, L-isoleucine, L-valine, L-phenylalanine, L-histidine, L-methionine, L-tryptophan, and L-lysine, significantly increased (*p* < 0.001) from day 0 to day 9. Moreover, several non-essential amino acids showed significant increases as well, including L-arginine, asparagine, glycine, L-aspartic acid, L-glutamic acid, and L-alanine. There is a lack of studies specifically addressing the increase in amino acids seen in *L. plantarum*-fermented apricot kernels. However, Song et al. [50] found that *L. plantarum* fermentation led to an increase in the total amino acid content of soybeans. The leucine, lysine, methionine, phenylalanine, and threonine levels in the fermented samples were higher than in raw ones. The most abundant amino acids in both raw and fermented soybeans were found to be aspartic acid and glutamic acid, the levels of which were significantly higher in the fermented samples. The aspartic acid levels increased from 4.29 g/100 g to 5.31 g/100 g, while the glutamic acid levels increased significantly from 6.66 g/100 g to 7.48 g/100 g.

### 3.6. Lactic Acid and Amygdalin Content

The fermentation significantly influenced the lactic acid production, with the levels peaking in the *L. plantarum*-fermented samples (LFSs) and no production being observed in the naturally fermented samples (NFSs). The amygdalin content decreased to undetectable levels as the lactic acid content increased, suggesting a role for *L. plantarum* in its metabolism. Detailed results are available in Supplementary Section S2.

### 3.7. The Relationship Between Polyphenols, Amino Acids and Antioxidant Activities

**Figure 3:**
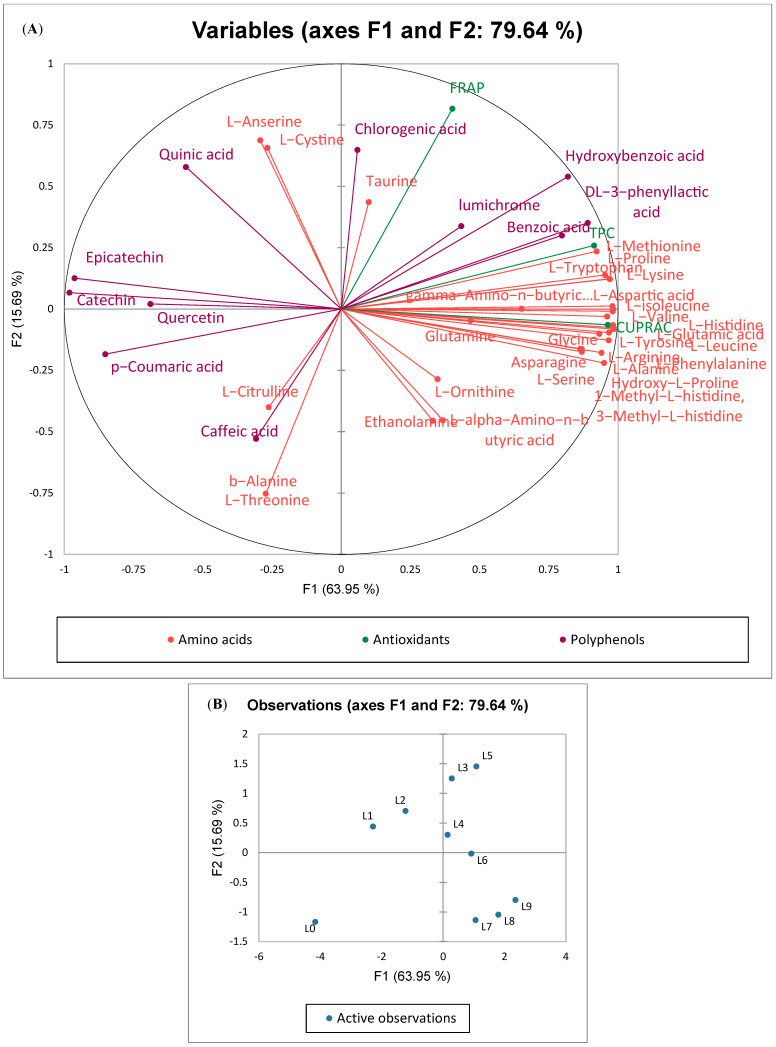
Multiple factor analysis (MFA) loadings (**A**) and score (**B**) plots correlating antioxidant values obtained using three different assays (FRAP, CUPRAC, and TPC) with phenolic compounds and amino acids observed during the fermentation of apricot kernels using *Lactiplantibacillus plantarum*.

Figure 3A,B illustrate the relationship of antioxidant activities (FRAP and CUPRAC) and the TPC with specific phenolic compounds and amino acids. The F1 axis explained 63.95% of the variance and distinguished the samples based on their varying fermentation times. The samples exposed to extended fermentation durations exhibited elevated positive scores along the F1 axis which corresponded with their CUPRAC antioxidant activity and TPC. The *L. plantarum*-fermented samples, on day 6, were correlated to the TPC, the CUPRAC antioxidant activity, most of the amino acids, and specific phenolic compounds including hydroxybenzoic acid, DL-3-phenyllactic acid, benzoic acid, and lumichrome. The samples fermented on days 7, 8, and 9 were associated with L-ornithine, L-alpha amino-n-butyric acid, and ethanolamine. Conversely, the samples fermented on days 1 and 2 exhibited significant negative scores along the F1 axis which were strongly connected with certain polyphenols such as catechin, epicatechin, p-coumaric acid, and quercetin. Factor 2 (F2) accounted for 15.69% of the variability and further distinguished the fermented seed samples that were fermented for 5 days, which exhibited a strong positive score along the F2 axis. These samples exhibited elevated FRAP antioxidant activity, along with the presence of chlorogenic acid and amino acids such as taurine, cystine, and anserine. Kuligowski et al. [10] fermented soybean seeds with four strains of *Rhizopus oligosporus* for 5 days. The antioxidant activity significantly increased until the fourth day, reaching a level almost 12 times higher than that of the unfermented sample. The highest level of TPC was recorded in soybean fermented for 4 days by the *R. oligosporus* strain NRRL 5905 (5.307 mg/g).

### 3.8. The Relationship Among Amino Acids in Terms of FRAP and CUPRAC Antioxidant Activities Using SHapley Additive exPlanations (SHAPs)

**Figure 4:**
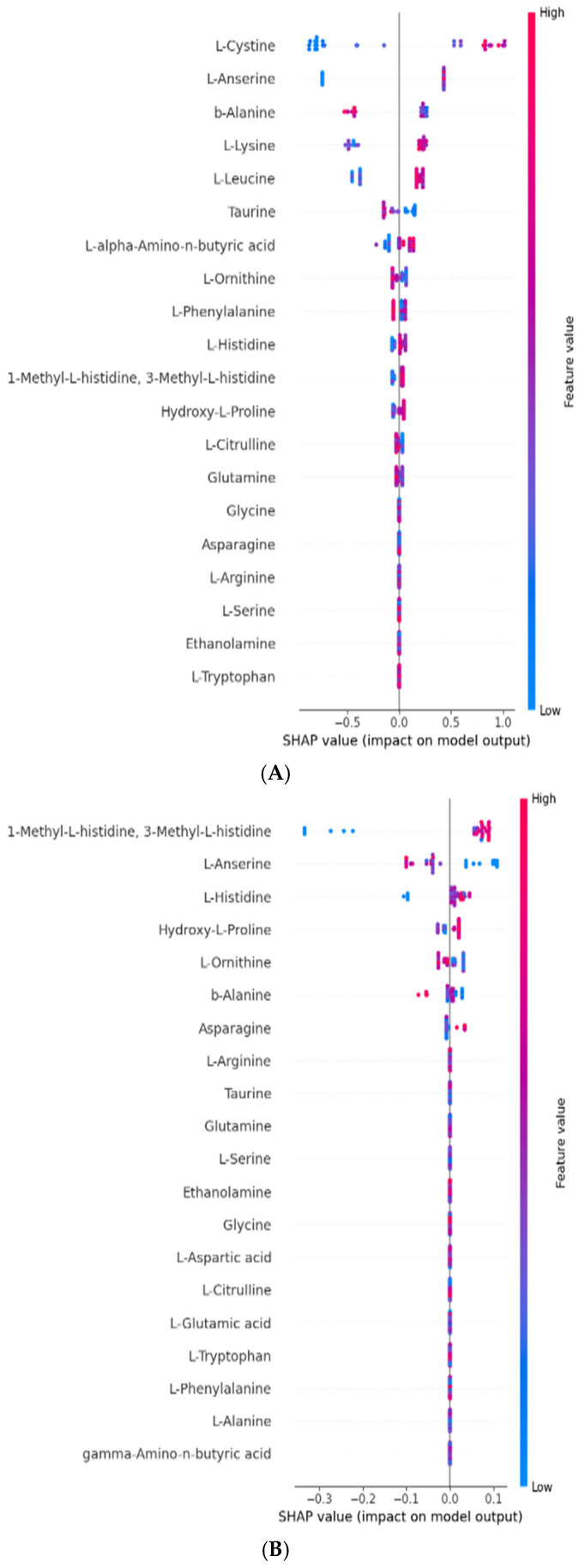
Magnitude and direction of the effects of the 30 amino acids on the antioxidant assays, (**A**) FRAP and (**B**) CUPRAC, indicated by SHAPs value. In each instance, the given explanation is represented by a single dot on each amino acid’s column. The dot colour is used to represent the relative value of the phenolic compounds. Blue represents lower polyphenol feature values. Purple represents intermediate polyphenol feature values and red represents higher feature values. The y-coordinate of the dot indicates the direction and magnitude of the effect of its amino acids’ concentrations on the SHAPs value.

**Figure 5:**
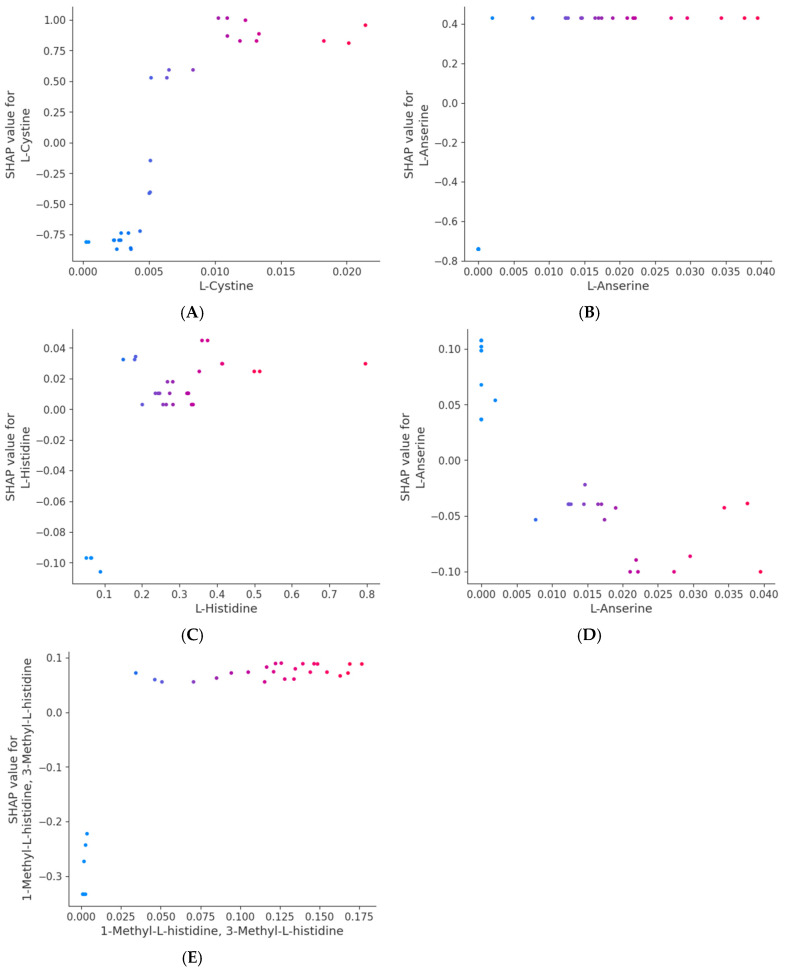
SHAPs-dependence plots displaying the top candidates for key amino acids that influence FRAP (**A**,**B**) and CUPRAC (**C**–**E**) antioxidant activities. The vertical axis represents the effect of each instance on the predicted value, while the horizontal axis represents the concentration of phenolic compounds. The colour of the dots indicates the concentration of the most strongly interacting phenolic compounds.

The SHAPs method is a method used to explain the output of machine learning models by providing feature importance scores for each input variable [51]. In this research, the SHAPs values were determined to explore the relationship between 30 amino acids identified during the fermentation of apricot kernels with *L. plantarum* and the FRAP and CUPRAC antioxidant activities, as seen in the SHAPs plots in Figure 4. The SHAPs analysis revealed that L-cystine and L-anserine were the most influential amino acids for the FRAP activity (Figure 4A). In Figure S1A, it was revealed that L-cystine and L-anserine demonstrated higher feature importance scores. This suggests a strong positive correlation of these amino acids with antioxidant activities, as determined by the FRAP assay and as is evident in the positive feature observed in Figure 5A,B. As L-cystine contains sulphur-containing side chains, it can be converted into reduced glutathione through redox cycles to neutralize free radicals, thereby providing protection against oxidative damage [52]. Conversely, L-anserine exhibited a negative correlation with the CUPRAC activity, likely due to its copper-chelating properties [53], which may decrease the availability of free copper ions, potentially diminishing or counteracting the antioxidant effect. This chelation effect could explain the negative feature observed in the CUPRAC measurement (Figure 5D). This highlights the importance of selecting complementary assays to fully capture antioxidant profiles.

Figure 4B illustrated the direction and magnitude of the impacts of various amino acids on the antioxidant capacity measured using the CUPRAC method. The plot shows that 1-methyl-L-histidine and 3-methyl-L-histidine had the most significant influence on the model output, with their high values (shown in red) contributing positively to the model output, indicating enhanced CUPRAC antioxidant capacity. On the other hand, low SHAPs values for histidine tended to negatively impact the model, indicating a reduced CUPRAC antioxidant capacity. Additionally, L-anserine and L-histidine also showed notable impacts, with L-histidine contributing positively at higher concentrations while the L-anserine had a negative impact at high concentrations. Amino acids like β-alanine and L-ornithine displayed smaller but still meaningful influences, suggesting their context-dependent roles in modulating CUPRAC values. Recent research by Zaky et al. [54] has also highlighted histidine and cysteine as amino acids with inherent antioxidant properties, further confirming their significance in antioxidant mechanisms. Anserine, which was found to be positively correlated to FRAP antioxidant activity, is a methylated derivative of carnosine and has been shown to be integral to antioxidant and anti-inflammatory processes [55]. Research has demonstrated its ability to inhibit lipid peroxidation within linoleic acid systems, functioning as a free radical scavenger, reducing agent, and copper ion chelator [53]. Furthermore, there is evidence suggesting that anserine supplementation is linked to elevated superoxide dismutase (SOD) levels, indicating an enhancement of antioxidant efficacy [56].

In Figure S1B, it can be seen that amino acids like L-histidine, 1-methyl-L-histidine, and 3-methyl-L-histidine exhibited high feature importance scores, suggesting a robust positive correlation with antioxidant activities, as evidenced in the CUPRAC assay and depicted in Figure 5C,E. Histidine significantly contributes to antioxidant activity by effectively giving protons to electron-deficient radicals, hence improving the radical-scavenging capabilities of bioactive peptides [57]. The FRAP and CUPRAC assays are related to this activity, as both assays are electron transfer-based assays that measure the capacity of an antioxidant in the reduction of an oxidant. The antioxidant capacity of histidine-containing peptides is linked to three key properties: their hydrogen donation, their lipid peroxyl radical trapping, and their metal ion chelation. These properties are conferred by the imidazole group, which is capable of donating hydrogen atoms to neutralise harmful free radicals and trapping lipid peroxyl radicals, which are particularly damaging to cell membranes [57]. Moreover, it has been suggested that the arrangement of peptides may also affect antioxidant function. Chen et al. [58] noted that replacing L-histidine with D-histidine in an antioxidative peptide diminished its efficacy. Their conclusion was that the accurate placement of the imidazole group is a critical determinant of antioxidant action.

### 3.9. The Relationship Among Phenolic Compounds in Terms of FRAP and CUPRAC Antioxidant Activities Using SHAPs Values

**Figure 6:**
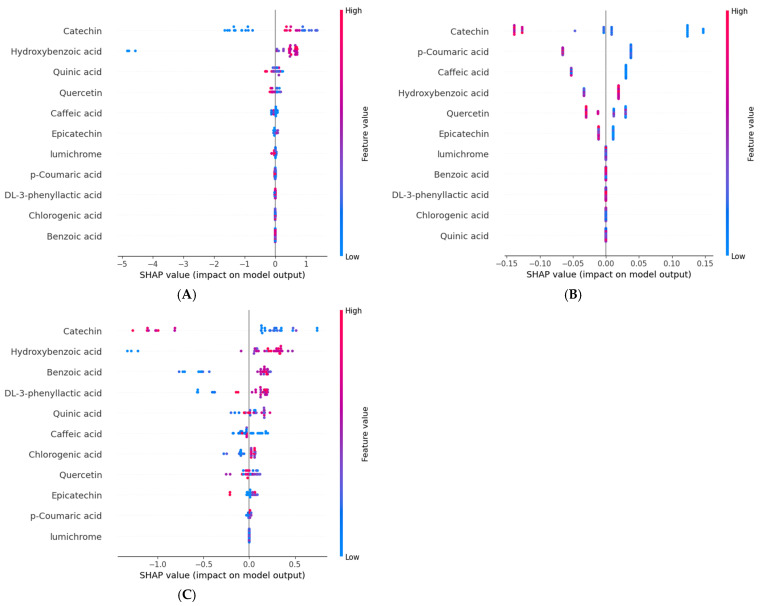
Magnitude and direction of the effects of the 11 phenolic compounds on the antioxidant assays and total phenolic content (TPC): (**A**) FRAP, (**B**) CUPRAC, and (**C**) TPC indicated by SHAPs values. In each instance, the given explanation is represented by a single dot on each polyphenol column. The y-coordinate of the dot indicates the direction and magnitude of the effect of its phenolic compound’s concentration on the SHAPs value.

SHAPs values were determined to identify the key phenolic compounds that contribute to antioxidant activities as assessed through the FRAP and CUPRAC assays, as well as the TPC, as depicted in Figure 6. Overall, specific phenolic compounds, particularly catechin and hydroxybenzoic acid, played critical roles in influencing the FRAP and CUPRAC antioxidant activities, as well as the TPC. The diverse and concentration-dependent effects of these phenolic compounds were essential in enhancing the antioxidant properties of the samples during fermentation, thereby emphasising their multifaceted contributions to the overall antioxidant capacity.

**Figure 7:**
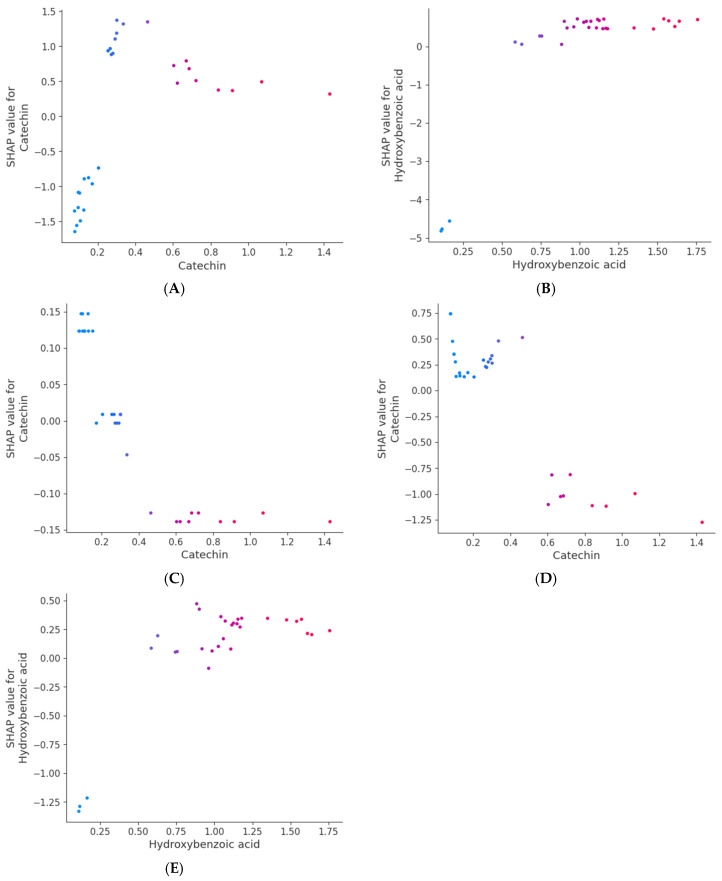
SHAPs-dependence plots displaying the top candidates for key phenolic compounds influencing FRAP (**A**,**B**) and CUPRAC (**C**) antioxidant activities as well as TPC (**D**,**E**). The vertical axis is the effect of each instance on the predicted value. The horizontal axis is the concentration of phenolic compounds. Colour of the dots indicates the concentration of the strongest interacting phenolic compounds.

Figure 6A illustrates the relationship between the levels of phenolic compounds and the SHAPs values for the FRAP antioxidant capacity. The results highlighted that catechin had the most significant influence on the model output and that the SHAPs values of hydroxybenzoic acid contributed positively to the FRAP antioxidant capacity. Figure S2A showed that catechin and hydroxybenzoic acid were the most important features, with the highest F scores of 84 and 62, respectively. Catechin had a significant positive impact on the model output at lower concentrations that were between 0 and 0.5 µg/g of sample, gently transitioning from negative to positive SHAPs values as the concentration increased (Figure 7A). However, when the concentration of catechin exceeds 0.5, the SHAPs values decreased. Catechin, a natural phenolic compound and antioxidant, significantly enhances the antioxidant capacity of various foods and beverages. The identified positive association between catechin and the antioxidant activity in the FRAP assay may be attributed to catechin’s capacity to donate electrons, promoting the reduction of ferric ions to ferrous ions, which underpins the FRAP assay [57]. Furthermore, Salman et al. [59] observed a substantial (*p* < 0.01) positive relationship (r^2^ > 0.9) between catechin and the antioxidant activity as determined by DPPH and ABTS assays. In addition, hydroxybenzoic acid exhibited a sharp increase in its SHAPs value at lower concentrations ranging from 0 to 0.5 µg/g and then stabilised at a high level until it reached a concentration of 1.75 µg/g, as depicted in Figure 7B.

In Figure 6B, the correlation between the concentration of phenolic compounds and the SHAPs values for the CUPRAC antioxidant capacity is presented. Figure S2B shows that the level of catechin had a significant impact on the model output, with high values generally decreasing the CUPRAC antioxidant capacity, and low values increasing it. This highlights a complex and concentration-dependent relationship between catechin and CUPRAC. A negative correlation was identified between catechin and the antioxidant activity using the CUPRAC assay (Figure 7C). Figure 7C demonstrates that, when the concentration of catechin exceeded 0.3 µg/g, the SHAPs value sharply declined and maintained a negative contribution until the catechin concentration reached its maximum. The decrease in catechin content during fermentation may account for its negative impact on the CUPRAC antioxidant activity. Tu et al. [60] discovered that the catechin content in green tea fermented with *Saccharomycodes ludwigii* at 25 °C for 120 h decreased significantly within the first 24 h, resulting in a 57.3% reduction in total catechins. This decline coincided with a notable increase in organic acids, particularly lactic acid. It is plausible that catechins could form reversible combinations with organic acids or other catechins in the highly acidic solution during fermentation, which may explain their reduced contribution to the CUPRAC antioxidant activity. As catechin’s ability to act as an electron donor was reduced during fermentation [57], its role in determining the antioxidant capacity, determined using the CUPRAC assay, was also diminished. In contrast, the levels of other phenolic compounds, such as DL-3-phenyllactic acid and benzoic acid, increased during fermentation (Figure 1), providing positive feedback in the assay. Additionally, the production of antioxidants like hydroxybenzoic acid [46] further contributes to the overall rise in the CUPRAC activity (Figure S3), compensating for catechin’s degradation and reinforcing its negative contribution to the total antioxidant capacity.

The relationships between phenolic compounds and their corresponding SHAPs values for TPC are shown in Figure 6C. The plot shows that catechin had a significant influence on the model output, with high values generally increasing the TPC, while low values were associated with a decrease, highlighting a concentration-dependent effect. Additionally, hydroxybenzoic was shown to have a significant positive impact on the TPC. Figure S2C further confirmed that catechin and hydroxybenzoic acid were significant contributors to the TPC, with the highest F scores of 63 and 61, respectively. However, a negative correlation was identified between catechin and the TPC (Figure 7D). The catechin SHAPs values remained relatively high at lower concentrations, ranging from 0 to 0.5 µg/g. However, as the concentration exceed 0.5 µg/g, the SHAPs values sharply decreased, eventually reaching negative values, until they reached a concentration of 1.4 µg/g. This may be attributed to the fact that the TPC reflects the total phenolic content of a sample, including not only catechin but also other phenolic compounds. According to Tu et al. [60], the levels of catechins in green tea fermented by *Saccharomycodes ludwigii* decreased rapidly by 29.6–47.6% during the first 24 h of fermentation and the total catechins decreased by 57.3% throughout the fermentation process. This change could be attributed to the drastic decrease in pH (from 5.16 to 2.31) that occurred during the fermentation, which led to the instability of catechins that in turn triggered degradation.

The positive correlation observed between hydroxybenzoic acid and the TPC is evident in Figure 7E, where sharp increases in the SHAPs values of hydroxybenzoic acid were observed at lower concentrations from 0 to 0.5 µg/g, with high SHAPs values being maintained thereafter until it reached a concentration of 1.75 µg/g. Moreover, this study identified this compound, alongside protocatechuic acid and ferulic acid, as a key contributor to the antioxidant capacity. Importantly, these phenolic acids were linked to the shikimic acid pathway, suggesting shared biosynthetic and regulatory mechanisms of their high contribution to the antioxidant activity. This proves the significant association identified among hydroxybenzoic acid, the total phenolic content, and the antioxidant capacity across various food matrices. Furthermore, Amarowicz et al. [61] exhibited elevated antioxidant activity in the acetone extract of faba beans, identifying hydroxybenzoic acid via LC-MS, which indicates that it contributes to enhancing the antioxidant activity in diverse legumes and further substantiating its effect on TPC.

### 3.10. Implications for Functional Food Development

The metabolic dynamics observed in this study suggest that optimising the fermentation duration (e.g., days 5–7) is critical to balancing the bioactive metabolite production and precursor stability. While prolonged fermentation enhances amino acid enrichment (e.g., L-histidine increased by 2.3-fold), it also risks depleting flavanol precursors (e.g., catechin undetectable by day 6). To avoid this, future studies could explore mixed-strain fermentation or sequential enzymatic treatments to preserve key antioxidants and maximize the metabolite yield. Such strategies align with the growing demand for naturally fortified ingredients in functional foods, as highlighted by the recent application of LAB-fermented cereals in gluten-free products [37].

## 4. Conclusions

This research was intended to improve the bioactivities of apricot kernels via fermentation, while investigating the relationship between both polyphenols and amino acids with antioxidant activities and the TPC by employing machine learning methodologies. The findings of this study indicate that the antioxidant activities, analysed using FRAP and CUPRAC assays as well as the TPC, significantly increased with an increase in the fermentation time. The hydroxybenzoic acid, catechin, epicatechin, DL-3-phenyllactic acid, and benzoic acid levels were found to be significantly affected by both the fermentation method and the time. As the fermentation time increased, there was a notable decline in the catechin and epicatechin content, while the content of benzoic acid, DL-3-phenyllactic acid, and hydroxybenzoic acid exhibited a notable increase. Furthermore, most amino acids were observed to increase in the sample fermented by *L. plantarum* with an increase in the fermentation time. Several amino acids and phenolic compounds exhibited a positive correlation with the antioxidant activities and TPC during the fermentation of apricot kernels using *L. plantarum*. While this study demonstrated the potential of *L. plantarum* to enhance the bioactivities of apricot kernels, certain limitations should be considered. As this study focused solely on one strain of *L. plantarum*, it remains unclear whether other *Lactobacillus* strains might yield similar results. Moreover, additional research is required to investigate the potential uses of fermented apricot kernels in the production of functional meals. Future investigations should specifically examine the durability of these bioactive chemicals throughout food processing and storage, along with their sensory attributes when integrated into food products. Additionally, in vivo studies should be conducted to assess the bioavailability and potential health benefits of these fermented products in humans.

## Figures and Tables

**Table 1:** Two-way analysis of variance with random effect on the results obtained from apricot kernels, which were naturally fermented (N) and fermented by *L. plantarum* (L). Significance codes: 0 < *** < 0.001. L indicates Lactobacillus, N indicates natural fermentation, and numbers 0–9 indicate days of fermentation. Different uppercase superscripts (A, B, C, D, E, F, G, H, I, J, and K) represent a statistically significant effect within the column.

Sample (Fermentation Method/Fermentation Time)	Microbial Colony Count (log cfu)	CUPRAC (mg Ascorbic Acid/g Sample)	FRAP (mg Ascorbic Acid/g Sample)	Total Phenolic Content (mg Gallic Acid/g Sample)	Lactic Acid Concentration (mg/mL)
L0	6.97 ± 0.08 ^FG^	1.03 ± 0.11 ^G^	4.90 ± 0.16 ^J^	1.67 ± 0.26 ^K^	0.11 ± 0.03 ^F^
L1	9.13 ± 0.10 ^C^	1.28 ± 0.00 ^F^	9.75 ± 0.10 ^D^	4.06 ± 0.23 ^GH^	8.01 ± 1.93 ^E^
L2	10.42 ± 0.10 ^B^	1.41 ± 0.11 ^EF^	10.75 ± 0.25 ^C^	4.84 ± 0.38 ^F^	16.25 ± 1.54 ^D^
L3	11.49 ± 0.23 ^A^	1.61 ± 0.11 ^CD^	12.20 ± 0.19 ^A^	7.15 ± 0.55 ^AB^	27.78 ± 0.64 ^B^
L4	8.77 ± 0.07 ^CD^	1.47 ± 0.06 ^DE^	11.43 ± 0.41 ^B^	6.65 ± 0.49 ^BC^	17.98 ± 1.45 ^D^
L5	8.29 ± 0.02 ^DE^	1.66 ± 0.08 ^BC^	11.27 ± 0.18 ^BC^	6.69 ± 0.54 ^BC^	36.70 ± 3.00 ^A^
L6	7.59 ± 0.130 ^EF^	1.57 ± 0.11 ^CD^	9.26 ± 0.37 ^DE^	5.63 ± 0.38 ^E^	25.72 ± 0.06 ^BC^
L7	6.34 ± 0.02 ^G^	1.82 ± 0.06 ^A^	8.88 ± 0.31 ^E^	6.58 ± 0.30 ^CD^	19.16 ± 2.70 ^D^
L8	5.33 ± 0.51 ^H^	1.79 ± 0.20 ^AB^	8.85 ± 0.38 ^E^	6.05 ± 0.35 ^DE^	21.10 ± 2.35 ^CD^
L9	4.39 ± 0.05 ^I^	1.79 ± 0.18 ^AB^	8.30 ± 0.44 ^F^	7.58 ± 0.38 ^A^	30.55 ± 0.53 ^AB^
N0	2.46 ± 0.15 ^J^	0.83 ± 0.03 ^H^	4.91 ± 0.34 ^J^	1.46 ± 0.32 ^K^	0.01 ± 0.00 ^F^
N1	2.76 ± 0.15 ^J^	0.67 ± 0.07 ^I^	4.51 ± 0.05 ^J^	2.34 ± 0.16 ^J^	0.00 ± 0.00 ^F^
N3	3.99 ± 0.03 ^I^	0.69 ± 0.04 ^HI^	5.80 ± 0.16 ^I^	3.14 ± 0.22 ^I^	0.00 ± 0.00 ^F^
N5	6.38 ± 0.26 ^G^	0.79 ± 0.04 ^HI^	7.06 ± 0.24 ^H^	3.84 ± 0.21 ^GH^	0.00 ± 0.00 ^F^
N7	7.74 ± 0.48 ^EF^	0.69 ± 0.01 ^HI^	7.12 ± 0.31 ^H^	3.74 ± 0.09 ^H^	0.00 ± 0.00 ^F^
N9	6.52 ± 0.86 ^G^	0.81 ± 0.05 ^HI^	7.71 ± 0.70 ^G^	4.34 ± 0.06 ^FG^	0.03 ± 0.01 ^F^
F value	36.923 ***	66.57 ***	169.87 ***	101.45 ***	35.495 ***

## Data Availability

The raw data supporting the conclusions of this article will be made available by the authors on request.

## Supplementary Materials

The following supporting information can be downloaded at: https://www.mdpi.com/article/10.3390/foods14050845/s1, Figure S1. Feature importance chart of amino acids for FRAP (A) and CUPRAC (B) antioxidant activities; Figure S2. Feature importance score of phenolic compounds for (A) FRAP, (B) CUPRAC and (C) TPC analyses; Figure S3. SHAP-dependence plot for the effects of Hydroxybenzoic acid in CUPRAC; Table S1. MRM transition for polyphenols; Table S2. MRM transition for AccQ-Tag amino acids; Table S3. Two-way analysis of variance of the response surface regression models obtained from the apricot kernels which were fermented by L. plantarum. A = Fermentation method (L or N), B = Fermentation time (0 to 9), * symbol represents p value (*p* *** < 0.001; 0.001 ≤ *p* ** < 0.01; 0.01 ≤ *p* * < 0.05; *p* ≥ 0.10); Table S4. Two-way analysis of variance of with random effect on the phenolic compounds concentration of sample obtained from the apricot kernels which were naturally fermented and fermented by L. plantarum: Signification codes: 0 < *** < 0.001 < ** < 0.01 < * < 0.05; L and N meant different fermentation method and number 0 to 9 meant fermentation time; Table S5. Two-way analysis of variance of with random effect on the amino acids concentration of sample obtained from the apricot kernels which were naturally fermented and fermented by L. plantarum: Signification codes: 0 < *** < 0.001 < ** < 0.01 < * < 0.05; L and N meant different fermentation method and number 0 to 9 meant fermentation time; Section S1. Microbial Analysis; Section S2. Lactic Acid and Amygdalin Content;
