# Supplementary material for: Improving the Bioactivities of Apricot Kernels Through Fermentation: Investigating the Relationship Between Bioactivities, Polyphenols, and Amino Acids Through the Random Forest Regression XAI Approach
Source: Foods. 2025 Feb 28;14(5):845. doi: 10.3390/foods14050845 (PMC11898452; doi:10.3390/foods14050845)
Supplement: Supplementary file 1 [file foods-14-00845-s001.zip › foods-3477207-supplementary.pdf]

Section S1: Microbial Analysis

Fermentation of apricot kernel particles was conducted using two approaches: natural fermentation (without inoculation) and fermentation with the inoculation of *L. plantarum*. The fermented samples were analysed in terms of the visual plate count values of lactic acid bacteria (LAB). The number of LAB within the apricot kernels showed a significant increase ($p < 0.05$) over a 9–day period during fermentation under both conditions, in comparison to the unfermented seed sample on day 0 (Table 1).

Table 1 showed a significant increase ($p < 0.001$) in the plate counts of LAB in *L. plantarum*–fermented samples during the first 3 days. Subsequently, there was a significant decline in colony counts observed from Day 4 to Day 9. Conversely, the LAB colony counts in naturally fermented samples remained relatively low during the initial fermentation period on Days 1 and 2. By Day 3, the colony numbers exhibited a significant increase, peaking on Day 7 before decreasing significantly by Day 9. These findings suggest that the apricot kernels inherently housed specific microorganisms that flourished under the fermentation conditions studied.

Section S2: Lactic Acid and Amygdalin Content

The results in Table 1 indicated that both the fermentation method and time had significant effects on lactic acid production. The two–way ANOVA results revealed that the fermentation method, time, and their interaction significantly influenced lactic acid concentration (F–value of 35.495; $P < 0.001$). Lactic acid concentration increased significantly ($P < 0.001$) with fermentation time in *L. plantarum*–fermented samples, reaching maximum levels on Day 5 and Day 7 of fermentation. In contrast, there was no lactic acid production observed in the naturally fermented samples. The increase in lactic acid concentration on Day 5 and Day 9 suggests active fermentation by the lactic acid bacteria. The increase is likely due to the optimal growth and metabolic activity of the bacteria, converting sugars present in the apricot kernels into lactic acid.

Amygdalin, a compound found in apricot kernels, poses a risk of toxicity due to its conversion to cyanide in the body upon ingestion. Ingesting more than three small raw apricot kernels can surpass safe levels and potentially lead to cyanide poisoning. The findings in this research showed a significant decrease in amygdalin content in apricot kernels with increasing fermentation time and lactic acid concentration. The initial level of amygdalin at 20 μg/mL on day 0 became undetectable on days 5 and 9 of fermentation as lactic acid levels increased.

Figure S1. Feature importance chart of amino acids for FRAP (A) and CUPRAC (B) antioxidant activities.

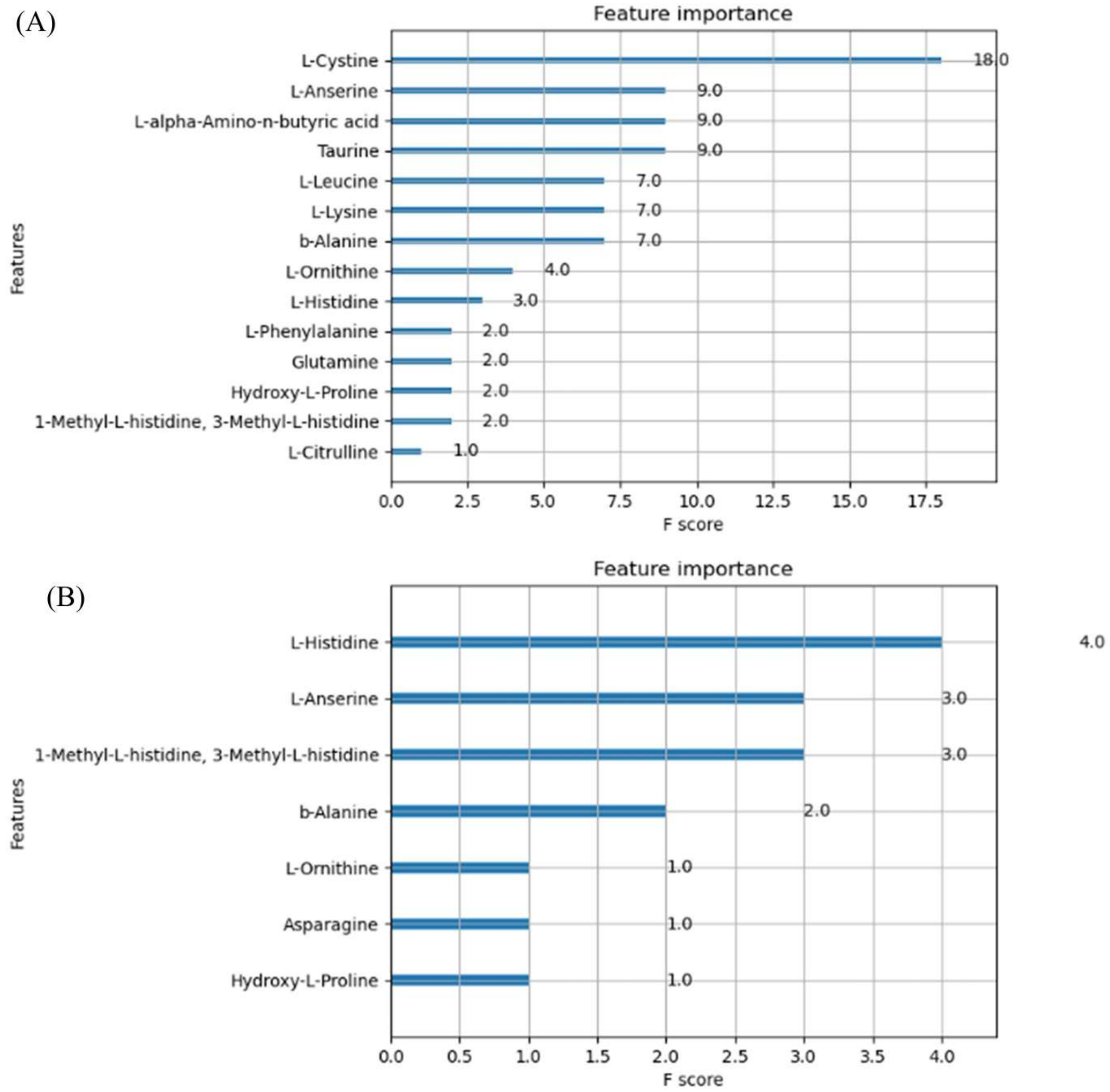

Figure S2. Feature importance score of phenolic compounds for (A) FRAP, (B) CUPRAC and (C) TPC analyses.

(A)

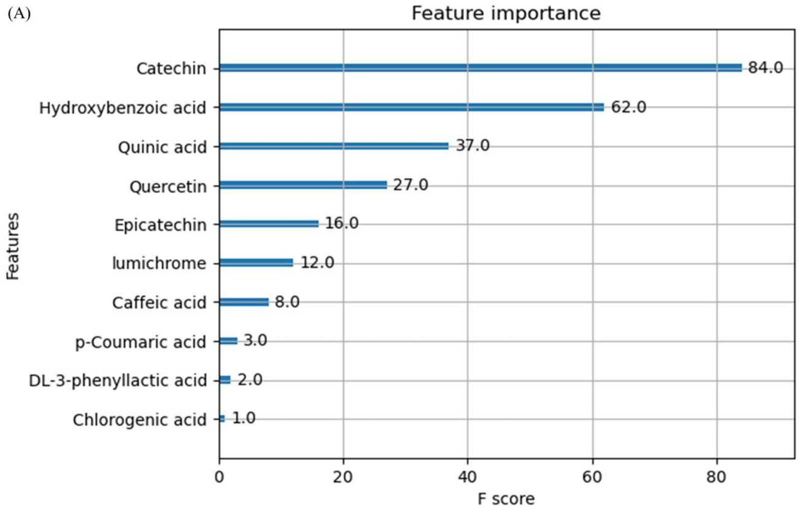

(B)

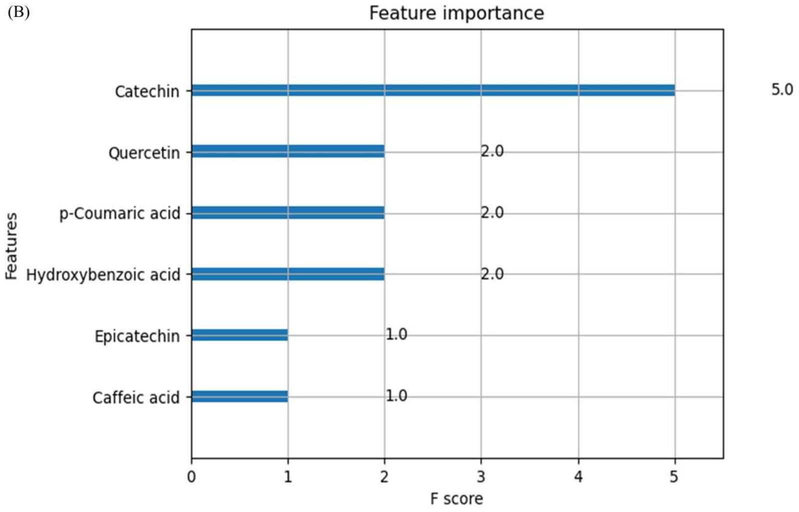

(C)

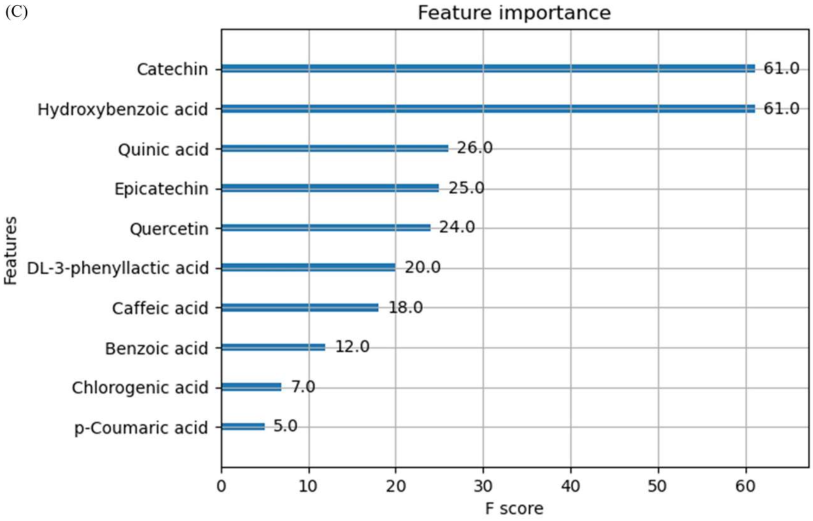

Figure S3. SHAP–dependence plot for the effects of Hydroxybenzoic acid in CUPRAC

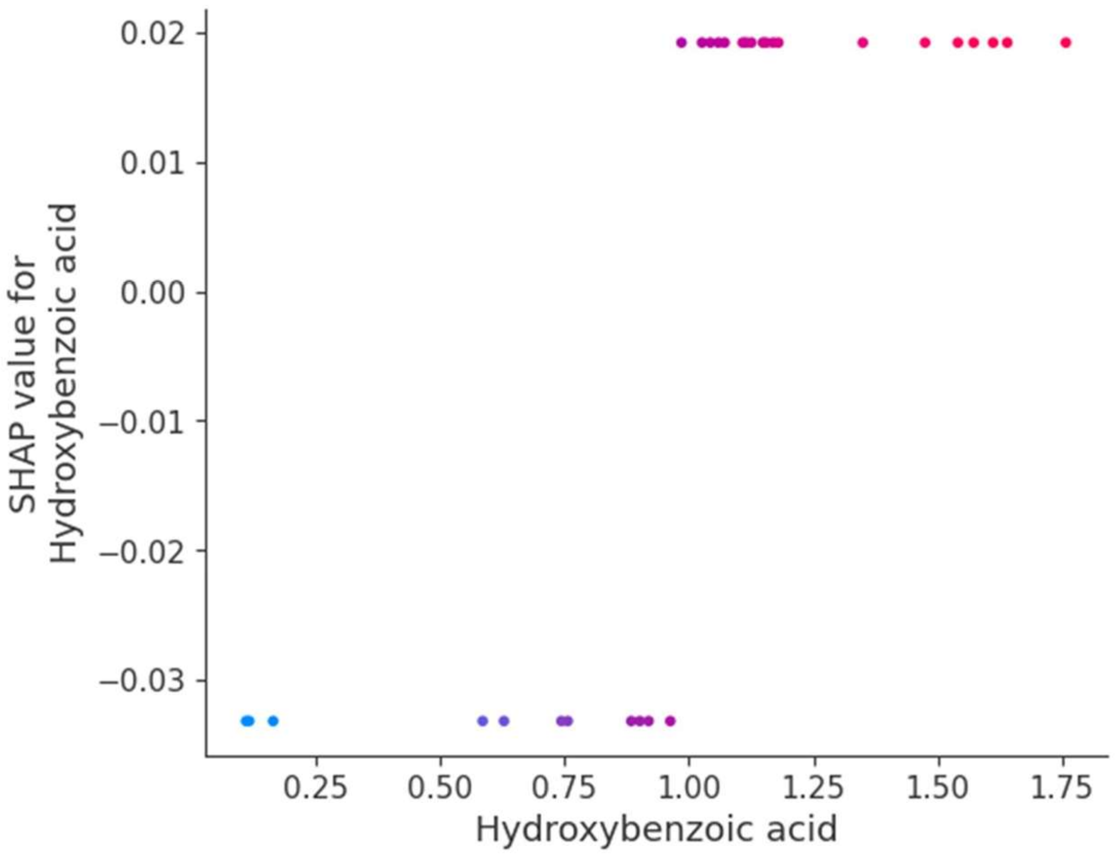

Table S1. MRM transition for polyphenols:

Compound Name	Precursor Ion	Product Ion	Fragmentor	Collision Energy	Cell Accelerator Voltage	Polarity
Benzoic acid	121	121	100	0	7	Negative
Benzoic acid	121	77	100	8	7	Negative
Caffeic acid	179	135	80	13	7	Negative
Caffeic acid	179	134	80	34	7	Negative
Catechin / Epicatechin	289	245	140	6	7	Negative
Catechin / Epicatechin	289	203	140	12	7	Negative
Chlorogenic Acid	353	191	80	13	7	Negative
Chlorogenic Acid	353	85	80	42	7	Negative
Hyrdoxybenzoic acid	137	137	80	0	7	Negative
p−Coumaric acid	163	119	80	12	7	Negative
p−Coumaric acid	163	93	80	36	7	Negative
Quercetin	301	179	140	10	7	Negative
Quercetin	301	151	140	16	7	Negative
Quinic acid	191	191	160	0	7	Negative
DL−3−phenyllactic acid	165	147	110	7	7	Negative
DL−3−phenyllactic acid	165	103	110	3	7	Negative
Lumichrome	241	241	100	2	7	Negative
Lumichrome	241	198	100	12	7	Negative

Table S2. MRM transition for AccQ–Tag amino acids.

Compound Name	Precursor Ion	Product Ion	Dwell	Fragmentor	Collision Energy	Cell Accelerator Voltage	Polarity
L–Citrulline	346	171	12	100	20	7	Positive
L–Arginine	345	171	12	100	20	7	Positive
1–Methyl–L–histidine, 3–Methyl–L–histidine	340	171	12	100	20	7	Positive
L–Histidine	326	171	12	100	20	7	Positive
L–Glutamic acid	318	171	12	100	20	7	Positive
L–Aspartic acid	304	171	12	100	20	7	Positive
Hydroxy–L–proline	302	171	12	100	20	7	Positive
Taurine	296	171	12	100	20	7	Positive
L–Threonine	290	171	12	100	20	7	Positive
L–Proline	286	171	12	100	20	7	Positive
L–Serine	276	171	12	100	20	7	Positive
gamma–Amino–n–butyric acid	274	171	12	100	20	7	Positive
d4–Alanine	264	171	12	100	20	7	Positive
Sarcosine	260	171	12	100	20	7	Positive
Glycine	246	171	12	100	20	7	Positive
Ethanolamine	232	171	12	100	20	7	Positive
L–Carnosine	397	171	12	100	20	7	Positive
L–Tyrosine	352	171	12	100	20	7	Positive
L–Methionine	320	171	12	100	20	7	Positive
L–Cystine	291	171	12	100	20	7	Positive
L–Valine	288	171	12	100	20	7	Positive
Cystathionine	282	171	12	100	20	7	Positive
L–alpha–Amino–n–butyric acid	274	171	12	100	20	7	Positive
delta–Hydroxylysine	252	171	12	100	20	7	Positive
L–Lysine	244	171	12	100	20	7	Positive
L–Ornithine	237	171	12	100	20	7	Positive
L–Anserine	411	171	12	100	20	7	Positive
L–Phenylalanine	336	171	100	100	20	7	Positive
L–Homocystine	305	171	100	100	20	7	Positive
L–Leucine	302	171	100	100	20	7	Positive
L–Tryptophan	375	171	100	100	20	7	Positive
Asparagine	303	171	12	100	20	7	Positive

Table S3. Two–way analysis of variance of the response surface regression models obtained from the apricot kernels which were fermented by L. plantarum. A = Fermentation method (L or N), B = Fermentation time (0 to 9), * symbol represents p value ($p^{***} < 0.001$; $0.001 \leqslant p^{**} < 0.01$; $0.01 \leqslant p^{*} < 0.05$; $p \geqslant 0.10$).

	Linear (F−value)		2−way interaction (F−value)
Compound	A. Fermentation method	B. Fermentation time	A*B
Quinic acid	4.06	16.64	1.09
Hyrdoxybenzoic acid	35.64***	55.73***	3.97
Catechin	33.54***	292.32***	13.09**
Chlorogenic acid	2.70	2.95	1.14
Caffeic acid	4.58*	0.87	0.16
Epicatechin	71.21***	423.97***	41.59***
DL−3−phenyllactic acid	120.86***	20.93***	19.30***
p−Coumaric acid	215.45***	3.37	9.00*
Benzoic acid	77.19***	13.86**	11.8**
Lumichrome	7.70**	6.21*	0.13
Quercetin	30.94***	0.95	2.47

Table S4. Two−way analysis of variance of with random effect on the phenolic compounds concentration of sample obtained from the apricot kernels which were naturally fermented and fermented by L. plantarum: Signification codes: 0 < *** < 0.001; L and N meant different fermentation method and number 0 to 9 meant fermentation time.

Category	Quinic acid	Hyrdoxybenzoic acid	Catechin	Chlorogenic acid	Caffeic acid	Epicatechin	DL−3−phenyllactic acid	p−Coumaric acid	Benzoic acid	Lumichrome	Quercetin
L0	18.959 def	0.516 g	4.248 a	1.394 cd	1.219 ab	0.129 a	0.423 f	0.450 d	45.333 de	0.119 cdefgh	0.003 a
L1	25.161 a	2.795 f	3.220 b	1.327 cde	0.779 ab	0.088 b	18.370 e	0.283 def	33.930 ef	0.084 fgh	0.002 abcd
L2	21.758 bcd	4.021 de	2.612 bc	1.322 cde	0.327 b	0.063 c	47.381 d	0.172 f	62.573 cd	0.081 gh	0.003 ab
L3	18.792 defg	4.908 cd	1.470 de	1.547 bc	0.171 b	0.044 d	71.418 c	0.204 f	99.445 b	0.183 abcdefg	0.002 bcdef
L4	17.546 efgh	4.356 cde	1.155 ef	1.249 cdef	0.312 b	0.038 d	54.454 d	0.242 ef	98.702 b	0.055 h	0.002 abcd
L5	20.914 cd	6.628 b	1.070 efg	2.129 a	0.193 b	0.035 d	99.620 a	0.189 f	138.132 a	0.286 a	0.003 abc
L6	17.677 efgh	4.910 cd	0.704 fghi	1.293 cde	0.177 b	0.000 e	74.391 bc	0.187 f	115.017 b	0.210 abcd	0.003 abc
L7	15.752 gh	3.530 ef	0.437 fghi	0.991 def	2.196 a	0.000 e	55.672 d	0.134 f	75.385 c	0.109 defgh	0.002 bcdef
L8	15.594 h	4.196 cde	0.445 fghi	1.356 cde	0.232 b	0.000 e	62.448 cd	0.171 f	95.842 b	0.119 cdefgh	0.002 abcdef
L9	17.618 efgh	5.339 c	0.328 hi	1.325 cde	0.151 b	0.000 e	87.211 ab	0.176 f	110.251 b	0.192 abcde	0.002 abcde
N0	20.498 cde	0.670 g	2.637 b	0.965 ef	0.910 ab	0.073 c	0.000 f	0.441 de	27.382 ef	0.101 efgh	0.001 def
N1	22.492 abc	4.215 cde	1.903 cd	1.563 bc	2.120 a	0.045 d	0.000 f	2.052 b	28.215 ef	0.174 bcdefg	0.001 f
N3	24.129 ab	8.127 a	1.039 efgh	1.853 ab	1.641 ab	0.000 e	0.000 f	2.516 a	35.726 ef	0.223 abc	0.001 cdef
N5	20.045 cdef	7.768 ab	0.423 ghi	0.964 ef	0.869 ab	0.000 e	0.328 f	2.125 b	37.052 ef	0.278 ab	0.001 ef
N7	16.968 fgh	6.616 b	0.219 i	0.838 f	0.874 ab	0.000 e	0.422 f	1.750 c	21.628 f	0.191 abcdef	0.001 f
N9	19.936 cdef	7.389 ab	0.088 i	1.030 def	1.079 ab	0.000 e	3.584 ef	2.096 b	37.742 ef	0.239 ab	0.001 cdef
F value	6.55***	31.20***	24.02***	5.49***	1.67***	79.71***	45.06***	164.68***	29.64***	3.69***	2.74***

Table S5. Two−way analysis of variance of with random effect on the amino acids concentration of sample obtained from the apricot kernels which were naturally fermented and fermented by L. plantarum: Signification codes: 0 < *** < 0.001 < ** < 0.01 < * < 0.05; L and N meant different fermentation method and number 0 to 9 meant fermentation time.

Category	L−Histidine	1−Methyl−L−histidine, 3−Methyl−L−histidine	Hydroxy−L−Proline	L−Arginine	Asparagine	Taurine	Glutamine	L−Serine	Ethanolamine	Glycine
L0	0.060 e	0.002 e	0.001 g	0.418 fg	1.468 defg	0.000 abc	0.323 c	0.473 f	0.053 bcde	0.094 h
L1	0.171 de	0.003 e	0.002 f	0.881 efg	1.626 cdefg	0.000 ab	0.695 bc	0.766 def	0.061 ab	0.337 fgh
L2	0.241 cde	0.056 d	0.001 f	0.936 def	1.740 cdef	0.000 abc	0.605 bc	0.882 cdef	0.058 abcd	0.486 def
L3	0.242 cde	0.088 c	0.002 ef	1.283 cde	1.879 bcde	0.000 abc	0.669 bc	0.970 bcdef	0.056 abcd	0.610 cdef
L4	0.330 bcd	0.136 a	0.002 ef	1.284 cde	2.031 abcd	0.000 bc	0.590 bc	1.025 bcde	0.063 ab	0.720 bcd
L5	0.332 bcd	0.105 bc	0.001 f	1.439 bcd	2.137 abcd	0.000 ab	0.615 bc	1.179 abcde	0.049 defg	0.817 abc
L6	0.311 bcd	0.137 a	0.002 def	1.710 abc	2.211 abc	0.000 abc	0.545 bc	1.209 abcde	0.053 bcdef	0.805 abc
L7	0.432 bc	0.132 ab	0.002 cd	1.227 cde	1.826 cdef	0.000 bc	0.375 bc	1.317 abc	0.060 abc	0.799 abc
L8	0.475 ab	0.152 a	0.002 bc	1.911 ab	2.578 ab	0.000 abc	0.586 bc	1.593 a	0.066 a	1.039 a
L9	0.306 bcd	0.160 a	0.002 cde	2.154 a	2.686 a	0.000 abc	1.772 a	1.411 ab	0.065 a	0.962 ab
N0	0.062 e	0.002 e	0.000 g	0.393 g	1.865 cde	0.001 a	0.402 bc	0.495 f	0.050 cdefg	0.120 gh
N1	0.438 b	0.003 e	0.002 ef	0.845 efg	1.145 fg	0.000 bc	0.690 bc	0.695 ef	0.043 efgh	0.370 efgh
N3	0.665 a	0.002 e	0.002 bc	0.999 de	1.767 cdef	0.000 c	1.316 ab	1.170 abcde	0.040 gh	0.614 cdef
N5	0.640 a	0.003 e	0.003 ab	1.127 de	1.809 cdef	0.000 c	0.826 abc	1.225 abcd	0.044 efgh	0.656 cde
N7	0.415 bc	0.001 e	0.003 a	0.755 efg	0.936 g	0.000 abc	0.466 bc	0.748 def	0.042 fgh	0.408 efg
N9	0.408 bc	0.003 e	0.003 a	0.878 efg	1.261 efg	0.000 abc	0.475 bc	0.892 bcdef	0.034 h	0.524 cdef
F value	6.62***	40.58***	21.45***	6.97***	3.73**	1.37	1.15	3.15**	6.87***	6.78***

Category	L−Aspartic acid	L−Citrulline	L−Glutamic acid	b−Alanine	L−Threonine	L−Alanine	γ −Amino−n−butyric acid	L−Proline	L−Ornithine	L−α −Amino−n−butyric acid
L0	0.149 h	0.008 bcd	1.071 h	0.008 a	0.503 c	0.726 f	0.820 d	0.322 fg	0.003 bcd	0.005 de
L1	0.630 fg	0.010 a	1.581 efgh	0.006 cde	0.406 cd	1.111 ef	0.717 d	0.638 ef	0.003 abcd	0.006 ab
L2	0.849 def	0.008 abcd	2.064 defg	0.006 cde	0.395 cd	1.485 cde	0.852 d	0.745 de	0.004 ab	0.005 abcde
L3	1.060 bcdef	0.006 defg	2.530 cd	0.006 def	0.374 cd	1.849 bcd	0.908 d	0.847 cde	0.003 abcd	0.005 cde

L4	1.036 bcdef	0.008 abc	2.586 bcd	0.006 bcd	0.424 cd	2.024 bc	0.881 d	0.855 cde	0.004 abc	0.006 abc
L5	1.302 abc	0.007 cdef	2.991 abc	0.005 ef	0.330 cd	2.296 ab	0.974 cd	0.993 bcd	0.003 d	0.005 de
L6	1.290 abcd	0.007 bcde	3.156 abc	0.005 def	0.356 cd	2.386 ab	0.917 d	0.933 cde	0.004 abc	0.005 bcde
L7	1.149 bcde	0.009 abc	2.457 cde	0.006 cde	0.392 cd	2.001 bc	0.754 d	0.796 cde	0.003 abcd	0.006 abcd
L8	1.622 a	0.009 ab	3.496 ab	0.007 ab	0.487 c	2.849 a	1.077 bcd	1.121 abc	0.004 ab	0.006 a
L9	1.485 ab	0.008 bcd	3.727 a	0.007 abc	0.455 cd	2.885 a	0.991 bcd	1.041 abcd	0.004 a	0.006 a
N0	0.258 gh	0.006 efgh	0.936 h	0.007 abc	0.216 d	0.720 f	0.825 d	0.246 g	0.003 cd	0.005 e
N1	0.651 fg	0.003 i	1.151 gh	0.005 f	0.839 b	1.192 def	1.341 ab	0.783 cde	0.001 e	0.002 g
N3	1.140 bcde	0.004 hi	2.070 def	0.005 f	1.307 a	1.773 bcde	1.555 a	1.334 ab	0.002 e	0.002 fg
N5	1.260 abcd	0.003 i	2.381 cdef	0.005 def	1.377 a	1.809 bcde	1.313 abc	1.363 a	0.001 e	0.003 f
N7	0.789 ef	0.004 ghi	1.515 fgh	0.005 ef	0.881 b	1.187 def	0.841 d	0.961 cde	0.001 e	0.003 fg
N9	0.938 cdef	0.005 fghi	1.732 defgh	0.005 f	0.986 b	1.342 cdef	0.879 d	1.102 abc	0.002 e	0.003 f
F value	6.78***	8.60***	7.21***	5.86***	16.32***	7.10***	3.38**	6.47***	10.99***	14.59***

Category	L−Lysine	L−Cystine	L−Anserine	L−Tyrosine	L−Methionine	L−Valine	L−isoleucine	L−Leucine	L−Phenylalanine	L−Tryptophan
L0	0.299 g	0.000 h	0.000 g	0.126 g	0.097 e	0.389 g	0.296 e	0.562 h	0.369 g	0.083 h
L1	1.001 def	0.017 bc	0.029 cd	0.722 f	0.419 d	1.085 f	0.876 d	1.661 gh	0.887 fg	0.363 g
L2	1.282 cde	0.016 bcd	0.030 cd	1.135 ef	0.479 cd	1.479 cdef	1.108 cd	2.361 efg	1.401 ef	0.458 efg
L3	1.606 abc	0.011 de	0.020 def	1.554 de	0.539 abcd	1.855 bcde	1.384 bc	3.107 cdef	1.996 de	0.551 cde
L4	1.531 bcd	0.009 ef	0.020 def	1.734 cd	0.535 abcd	1.968 bc	1.492 bc	3.499 cde	2.405 cd	0.605 bcd
L5	1.823 abc	0.006 fg	0.009 fg	1.997 bcd	0.575 abcd	2.316 ab	1.737 ab	4.037 bc	2.747 bc	0.617 abcd
L6	1.795 abc	0.004 fgh	0.012 efg	2.202 bc	0.574 abcd	2.410 ab	1.746 ab	3.871 bcd	3.057 bc	0.659 abc
L7	1.484 bcd	0.003 gh	0.000 g	1.812 cd	0.477 cd	1.980 bc	1.485 bc	3.664 bcd	2.505 cd	0.553 cde
L8	2.135 a	0.003 gh	0.006 fg	2.450 ab	0.658 ab	2.703 a	2.078 a	4.849 ab	3.304 ab	0.703 ab
L9	2.010 ab	0.004 fgh	0.000 g	2.750 a	0.644 abc	2.859 a	2.152 a	5.406 a	3.928 a	0.729 a
N0	0.186 g	0.000 h	0.000 g	0.188 g	0.150 e	0.358 g	0.283 e	0.472 h	0.330 g	0.156 h
N1	0.893 ef	0.015 bcd	0.034 bcd	0.890 f	0.537 abcd	1.304 ef	1.077 cd	2.021 fg	1.104 f	0.414 fg
N3	1.619 abc	0.013 cde	0.023 de	1.206 ef	0.683 ab	1.897 bcde	1.498 bc	2.684 defg	1.438 ef	0.546 cde
N5	1.674 abc	0.019 ab	0.042 abc	1.225 ef	0.700 a	1.923 bcd	1.543 bc	2.689 defg	1.377 ef	0.521 def
N7	0.644 fg	0.022 a	0.054 a	0.859 f	0.474 d	1.334 def	1.069 cd	1.893 g	1.044 f	0.413 fg
N9	0.588 fg	0.024 a	0.047 ab	0.971 f	0.524 bcd	0.945 fg	1.143 cd	2.027 fg	1.146 f	0.395 g
F value	9.32***	20.62***	13.15***	18.53***	8.51***	12.18***	10.31***	11.20***	22.18***	19.23***
